# The use of nutrient-optimizing/cost-minimizing software to develop ready-to-use therapeutic foods for malnourished pregnant women in Mali

**DOI:** 10.1002/fsn3.175

**Published:** 2015-01-21

**Authors:** Allison Bechman, Robert D Phillips, Jinru Chen

**Affiliations:** Department of Food Science and Technology, The University of Georgia1109 Experiment St., Griffin, Georgia, 30223-1797

**Keywords:** Linear programming, malnutrition, ready-to-use therapeutic foods

## Abstract

Malnutrition affects people of all ages in many countries in the developing world. One treatment for malnutrition is the intervention involving ready-to-use therapeutic foods (RUTFs). This study developed RUTFs for pregnant women in Mali using formulation computer software and largely local, plant-based ingredients. Mali has the world's second highest birth rate and infant mortality rate. Nutrient profiles of possible ingredients and their prices from 2004 to 2009 were entered into the software. Computer-selected ingredients included peanuts, cowpeas, and millet as well as rice or barley koji (sources of *α*-amylase and ingredients). Components of the six selected formulations were milled, hydrolyzed with koji *α*-amylase, and heated at 121°C for 15 min. The contents of protein, fat, ash, fiber, carbohydrates, amino acid, and energy of dehydrated products were determined and compared with software-predicted values. Actual and predicted values were comparable: the protein content was 1.45–2.04% higher, and ash content was 0.60–0.89% higher than the predicted values, while the fat content was 0.18–0.88% lower, the lysine content was 0.17–0.25% lower, and fiber content was 0.16% lower to 2.06% higher than the predicted values. The difference in actual and predicted energy levels were 14.8–22.2%. The amount of RUTF needed to meet the requirement of most limiting nutrients, lysine and energy, ranged from 2620 to 3002 g. The costs for producing the RUTFs were substantially lower than importing commercial RUTFs even with increased ingredient prices in Mali from 2004 to 2009.

## Introduction

A healthy population is one of the country's most valuable resources, as healthy citizens are able to contribute to both society and the economy. However, acute diseases and chronic conditions may threaten the health of individuals and large segments of the population, especially in developing countries. Malnutrition, which results from diets that do not provide sufficient energy and essential nutrients, is a chronic condition that impacts much of the developing world (Briend and Nestel 2005).

While malnutrition is commonly associated with children, people of all ages, especially pregnant women, are susceptible. Malnourished pregnant women exhibit greater maternal morbidity—infections and anemia—and mortality compared to healthy individuals (Lartey 2008). During pregnancy, malnutrition also threatens the health of their unborn children (NHD/SDE/WHO 2001). Malnourished pregnant women are at a greater risk of giving birth to infants who are smaller, weaker, less resistant to disease, have a higher mortality rate, may be less intelligent, and have a higher rate of impaired physical development than those born to well-nourished mothers (Belli 1971; Victora et al. 2008). These babies are very likely to become malnourished adults themselves, in an intergenerational cycle of malnutrition (Briend and Nestel 2005).

One successful treatment for malnutrition in children is the intervention involving ready-to-use therapeutic foods (RUTFs). RUTFs are energy- and macronutrient-dense foods fortified with vitamins and minerals (Collins et al. 2006). Most existing RUTFs are made of peanuts, powdered sugar, oil, powdered milk, vitamins, and minerals (Nutriset 2012). An important advantage of RUTFs, other than their nutritional benefit, is that they require no preparation after processing, allowing for home treatment of moderate cases of malnutrition, rather than hospitalization (Linneman et al. 2007).

However, most current RUTFs contain powdered milk, which is not commonly available in most developing countries, making the RUTFs expensive to produce and difficult to access by poor, malnourished populations (Manary 2006; Dibari et al. 2012). These products also contain a high percentage of peanut paste which has a thick consistency, making them difficult for some individuals to swallow (Manary 2006). Research has shown that thickened nutritious beverages are better alternatives for patients who have difficulty in eating and swallowing since the products hydrate the oral cavity and reduce the speed of liquid flow through the digestive tract (Germain et al. 2006).

Utilization of local ingredients to produce a RUTF in a country or region should reduce the total cost. The food staples in West Africa include cereals, legumes, oilseeds, and starchy roots and tubers which must be combined in proper proportions to provide the nutrients pregnant women need (FAO 2011). To produce favorable formulations while minimizing costs, it is convenient to use mathematical models, such as linear programming techniques that are employed in developing rations for the animal feed industry (Udo et al. 2011; Dibari et al. 2012). Linear programming has also been used in human nutrition since 1959 (Smith 1959) and can be used to assess the economic value of fortified food supplements and predict limiting nutrients in a developed formulation, making it a suitable tool for the development of RUTFs (Dibari et al. 2012).

Most available RUTFs have been designed for treatment of malnutrition in children and are not optimal for pregnant women who have different nutritional requirements. In general, the nutritional status of West African women is poor, with 13–20% of women of childbearing age having a body mass index (BMI) indicative of chronic energy deficiency (Lopriore and Muehloff 2003). The objective of this research was to develop low-cost, plant-based RUTFs targeting malnourished pregnant women in Mali, a West African country with the second highest birth rate and infant mortality rate in the world (CIA 2011).

## Materials and Methods

### Ingredients and their nutrient profile and cost

Corn, sorghum, peanuts, millet, fonio, cassava, cowpeas, rice, barley, yams, sugar, and sesame were chosen as potential ingredients of the RUTFs. These ingredients were selected based on the foods commonly consumed and the agricultural commodities with the highest production rates in Mali (Torheim et al. 2004; United States Agency for International Development 2009; Aly et al. 2011; FAO PRODUCTION STAT 2013). Representative nutrient profiles of all ingredients were obtained from the USDA (2012) except for the profile of fonio, which was from a journal article (Clottey et al. 2006). The prices of all ingredients, except sugar, during the period 2004–2009 were obtained from FAO PRICE STAT (2013) and are displayed in Table1. A single price of sugar was obtained from a Malian newspaper article (Table1) (Le Mali En un Clic JournalduMali.com 2009).

**Table 1:** Prices for potential RUTF ingredients ($USD/kg) over a 6-year period in Mali^1^

	Ingredient										
Year	Corn	Sorghum	Millet	Barley^2^	Rice	Peanuts	Cowpeas	Yam	Cassava	Fonio	Sugar^3^
2004	$0.10	$0.11	$0.12	$0.19	$0.21	$0.24	$0.26	$0.34	$0.38	$0.56	$1.03
2005	$0.20	$0.23	$0.25	$0.19	$0.27	$0.24	$0.26	$0.63	$0.38	$0.56	$1.03
2006	$0.17	$0.18	$0.20	$0.19	$0.25	$0.31	$0.26	$0.63	$0.38	$0.57	$1.03
2007	$0.15	$0.18	$0.18	$0.22	$0.25	$0.40	$0.30	$0.31	$0.11	$0.63	$1.03
2008	$0.17	$0.19	$0.19	$0.23	$0.26	$0.47	$0.36	$0.28	$0.09	$0.83	$1.03
2009	$0.18	$0.24	$0.29	$0.34	$0.32	$0.51	$0.35	$0.23	$0.06	$0.83	$1.03

1 FAO PRICE STAT (2013).

2 Price data on barley from Mali were not available, so data from Algeria, a African country bordering Mali, were used.

3 One price for sugar was found for Mali based on a 2009 newspaper article (Le Mali En un Clic JournalduMali.com 2009).

Along with identifying potential ingredients for RUTFs, an enzyme source was also needed in processing the RUTFs. Rice and barley fermented with *Aspergillus oryzae* (termed rice and barley koji) were made and used in the production of the RUTFs. Koji usually contains several enzymes including *α*- and *β*-amylase, glucoamylases, *α*-glucosidase, and acid and neutral proteases (Chou and Rwan 1995). Amylase activities of the koji used in this study were determined by the 3, 5-dinitrosalicylic acid method as described by Miller (1959) with modifications. Each gram of rice or barley koji used in the present study had 59.78 or 117.46 U (expressed as mg maltose released/g koji solids/min) of *α*-amylase, respectively (Bechman et al. 2012). Protease contents of the rice and barley koji were found to be very low (Bechman et al. 2012). The amylase were used to hydrolyze the starch in the products, reducing the thickness of the RUTFs, making it easier to swallow and making the nutrients easier to be absorbed by malnourished individuals. However, the nutrient composition of the fermented grains was not available, and therefore the nutrient profile of unfermented grains was used.

### Computer software and product formulation

Formulation computer software was used to develop the RUTFs (CFC4-S2®; Creative Formulation Concepts, LLC, Annapolis, MD). The software utilized linear equations to develop a formulation optimized for the nutrient profile desired while minimizing the cost. All potential ingredients and their nutrient profiles and prices were entered into the software. The nutrient requirements of pregnant women in their third trimester set by the Food and Agriculture Organization and the Institute of Medicine/US National Academy of Sciences (Dietary Reference Intakes) were used as references to develop the RUTFs (Table2) (FAO, WHO, UNU 2001; Food and Nutrition Information Center 2011).

**Table 2:** Daily nutrient requirements and formulation restrictions for macronutrients and amino acids utilized in the development of the RUTFs

Nutrient	Amount
kcal/day^1^	2615.20
Protein (g/day)^1^	64.00
Carbohydrate (g/day)	65.00
Fat (g/day)^2^	58.12
Amino acids (g/day)^3^	
Lys	3.26
Leu	3.52
Val	2.05
His	1.15
Trp	0.45
Thr	1.73
Ile	1.60
Met + Cys	1.60
Phe + Tyr	3.01

1 FAO, WHO, UNU (2001).

2 Food and Nutrition Information Center (2011).

3 Amino acids requirements calculated using the amino acid scoring pattern for 1- to 3-year-old children; Food and Nutrition Information Center (2011).

The formulation software allows restrictions to be placed on both the ingredients and the level of nutrients used in the RUTFs. Restrictions placed on nutrients helps ensure that the RUTFs provide necessary nutrition to the target population in each serving. Restrictions on the ingredients can be used to improve the palatability of the products by limiting excess amounts of certain ingredients when necessary. In the present research, restrictions were placed on nutrients and ingredients in order to develop nutritionally desirable RUTFs. Table2 shows the nutrient restrictions applied in this optimization. A maximum restriction of 20% was set for the total fat content based on the Acceptable Macronutrient Distribution Range (Institute of Medicine of the National Academies 2005) (Table2). A minimum of 452 kcal/100 g was specified to ensure a high energy content, and minimum levels of essential amino acids based on the reference pattern for 1- to 3-year-old children were specified. Daily amino acid requirements for pregnant women were unavailable (FAO, WHO, UNU 2001; Food and Nutrition Information Center 2011), therefore, the amino acid scoring pattern for 1- to 3-year-old children was used. Like toddlers, pregnant women have high nutrient requirements due to increased physiologic demands, and therefore, formulation of RUTFs with this approach will provide a sufficient level of amino acids for the target population (Food and Nutrition Information Center 2011). Based on the results of preliminary formulations, sugar was restricted to a maximum level of 14%. No restrictions were placed on micronutrients because a vitamin/mineral premix will later be added to these base formulations to make complete RUTFs. Restrictions were also placed on the amount of rice (7, 14, or 21%) or barley (5, 10, or 15%) koji used in the products based on the amount of *α*-amylase needed to breakdown at least 50% of the starch in the RUTFs during processing.

Once the 12 local ingredients from Mali and necessary information were entered into the software, formulations were developed using the ingredient price data during the period of 2004 to 2009. Based on the nutrient requirements, restrictions described above, and ingredient cost, six base RUTF formulations consisting of peanuts, cowpeas, millet, and rice or barley (Table3) were selected for processing and characterization.

**Table 3:** RUTF formulations generated using creative concepts formulation software

	Formulations with rice koji			Formulations with barley koji		
Ingredients (%)	A	B	C	D	E	F
Peanuts	38.4	39.0	39.5	38.2	38.5	38.8
Cowpeas	22.2	21.8	21.4	21.9	21.2	20.5
Millet	18.4	11.2	4.1	20.9	16.3	11.8
Sugar	14.0	14.0	14.0	14.0	14.0	14.0
Rice	7.00	14.0	21.0	–	–	–
Barley	–	–	–	5.00	10.0	15.0

### Processing of RUTFs

Based on the restrictions described in Table2 and the ingredient prices (Table1), 6 RUTF products were formulated and processed. The selected ingredients for the six formulations (Table3) were obtained from local sources: blanched, roasted peanuts from American Blanching Company (Fitzgerald, GA), cowpeas and millet flour from Dekalb Farmers Market (Decatur, GA), rice and barley from Sevananda Natural Foods Market (Atlanta, GA), and sugar from local retail stores (Griffin, GA). Decorticated cowpeas and millet flour were boiled with tap water (1:10) separately, and then peanuts were added into the mixture. Rice (7, 14, or 21% of the formulation) or barley (5, 10, or 15% of the formulation) was added in the form of koji, serving both as an ingredient and a source of *α*-amylase (Bechman et al. 2012). After the addition of koji, water (72–245 mL) was added and diluted mixtures were passed two times through a colloid mill (Morehouse Industries, Los Angeles, CA). The quantity of additional water added was determined by subtracting the water used for boiling the cowpeas and millet flour from the total batch size of the product. The milled product was divided into eight-quart stainless steel cooking pots (Crate and Barrel, Northbrook, IL), covered with glass lids and held at 55°C for 4 h in a reciprocal shaking water bath at 50 rpm (ThermoScientific, Marietta, OH) to allow the *α*-amylase to hydrolyze the starch. After 4 h, 6 Gelatin Digesting Units/g of bromelain (Kalyx, Camden, NY) was added to the RUTF mixture. Following the addition of bromelain, the RUTFs were incubated for 30 min in the water bath at 55°C and 50 rpm. The product was then boiled for 10 min on a stove (Amana, Benton Harbor, MI) to inactivate enzymes, followed by the addition of sugar and salt (Wal-mart, Griffin, GA). The product was filtered through a 2-mm sieve (Fisher Scientific, Pittsburgh, PA), filled into 160 mL milk dilution bottles (Fisher Scientific), autoclaved at 121°C for 15 min (Steris, Mentor, OH), cooled, and stored at 4°C until analysis.

### Nutrient analysis

After processing, subsamples of the RUTFs were frozen at −20°C and freeze-dried for 18 h at 20 ± 2°C (The Virtis Company Inc, Gardiner, NY) in plastic containers (5.5 × 5.5 × 2 in; Rubbermaid, Atlanta, GA) covered with aluminum foil (Fisher Scientific; with punched holes). Protein analysis was performed using the combustion method (AOAC 2000). Amino acid profiles of the RUTFs were analyzed using acid hydrolysis, followed by derivatization, and separation using high-performance liquid chromatography (Covance Laboratories 2012). Total fat content was determined using the Goldfisch extraction method (948.22; AOAC 2000). Ash contents were measured using the incineration method (Ba 5–49; AOCS 1998). Moisture contents were analyzed using the vacuum oven drying method (925.10; AOAC 2000), and total dietary fiber was measured using the enzymatic-gravimetric method (985.29; AOAC 2000). Total carbohydrate was calculated by difference, and the energy (kcal) was calculated as the sum of the total amount of protein, fat, and carbohydrate (g) in an individual RUTF multiplied by the amount of energy provided by a unit dry weight of each nutritional component (4 kcal/g of protein and carbohydrates; 9 kcal/g of fat) (FAO 2003). Based on obtained nutrient values and the assumption that the RUTFs are the only source of nutrients, the amount of RUTFs that has to be consumed in order to meet the daily nutrient requirements of a pregnant woman was determined. The daily requirement for each nutritional component was divided by the actual nutrient content in unit dry weight of the RUTFs.

### Statistical analysis

Data were analyzed using Statistical Analysis Software (version 9.1; Cary, NC). Two-way analysis of variance (ANOVA) and Fisher's least significant difference test were used to determine the significant differences based on a confidence level of 95%.

## Results and Discussion

### Formulation and analysis of computer-generated RUTFs

The amounts of peanut and cowpea in the six selected RUTF formulations were similar (Table3). However, millet flour contents varied inversely with restricted rice and barley koji levels (Table3), with one cereal displacing the other.

Predicted and measured contents of macronutrients were reasonably close as shown in Table4. Actual protein contents of the products were 1.45–2.04% higher, while the actual fat contents were 0.18–0.88% lower than the predicted values (Table4). Differences between predicted and actual ash contents were 0.60–0.89% (Table4). The fiber content of the RUTFs containing rice koji varied from −0.16% to 0.49% from predicted values, while that of the barley koji-containing RUTFs was 0.92–2.06% lower than the predicted values. Greater differences (14.8–22.2%) were noticed between predicted and actual energy contents of the RUTFs (Table4).

**Table 4:** Software-predicted and actual macronutrients of the RUTFs (on a dry weight basis)

	Formulations with rice koji				Formulations with barley koji			
Nutrient	Product	Predicted values	Actual values	Absolute difference	Product	Predicted values	Actual values	Absolute difference
Protein (%)	A	17.6	19.5	1.86	D	17.8	19.8	2.04
	B	17.3	19.3	1.95	E	17.7	19.6	1.92
	C	17.1	18.5	1.45	F	17.6	19.6	1.99
Fat (%)	A	20.0	19.3	−0.68	D	20.0	19.8	−0.19
	B	20.0	19.1	−0.88	E	20.0	19.8	−0.18
	C	20.0	19.2	−0.86	F	20.0	19.6	−0.43
Ash (%)	A	2.25	2.95	0.70	D	2.34	2.94	0.60
	B	2.06	2.81	0.75	E	2.23	2.91	0.68
	C	1.87	2.76	0.89	F	2.12	2.89	0.77
Fiber (%)	A	7.18	6.90	−0.28	D	8.13	6.07	−2.06
	B	6.58	6.42	−0.16	E	8.47	7.16	−1.31
	C	5.97	6.46	0.49	F	8.81	7.89	−0.92
Carbohydrate (%)	A	52.5	51.3	−1.13	D	52.4	51.3	−1.10
	B	52.7	52.4	−0.32	E	52.6	50.5	−2.12
	C	52.9	53.1	0.25	F	52.8	50.1	−2.72
Energy (kcal/100 g)	A	441	457	16.0	D	441	463	22.2
	B	441	459	17.5	E	440	459	18.4
	C	441	459	17.7	F	440	455	14.8

Observed variations in predicted and actual values were probably due to the differences between reference and actual nutrient values of the ingredients used in the study. The USDA nutrient data on the RUTF ingredients were based on raw, whole commodities (Clottey et al. 2006; USDA 2012). Different forms of ingredients were, however, used in the present study: the cowpeas were decorticated, peanuts were roasted, rice and barley were fermented, and millet flour instead of millet seed was used. Furthermore, the nutrition profiles of the RUTF products reported in the present study were determined after processing and dehydration which would have impacted the nutritional composition of the products (Table4).

El-Habashy et al. (1995) also found a close agreement between actual and predicted protein content of weaning foods developed using formulation software (0.4% lower, 2.1% higher); fat content (1.1–1.7% higher); and the ash content (0.1–0.5% higher). A RUTF developed by Dibari et al. (2012) for malnourished adults with HIV had an energy content that was 15.76% higher, protein content 2.3% higher, and a fat content 1.0% lower than predicted values. These findings, in total, confirm that optimization software is a valuable tool for generating formulae meeting specific nutritional requirements.

The commercially available RUTF often used in the treatment of malnutrition, specifically in children, PlumpyNut®, is made of peanut butter, sugar, oil, and nonfat dry milk (NFDM), along with a vitamin/mineral premix (Therapeutic Food 2012). PlumpyNut® contains 545 kcal, 13.6 g protein, and 35.7 g fat per 100 g (Therapeutic Food 2012). The present RUTFs contain ∽1.30 to 1.50-fold more protein, ∽1.80-fold less fat, and 1.18 to 1.20-fold less energy than PlumpyNut® (Therapeutic Food 2012). Difference in fat and subsequently energy contents between the current RUTFs and PlumpyNut® is that the latter contains approximately 15% vegetable oil (Manary 2006). The fat content of the present RUTFs was restricted to balance the overall nutrition profile of the formulations to meet the needs of malnourished pregnant women. The fat content in the formulations could be raised, by the addition of oil for example, which would result in a higher fat and energy content without compromising the availability of other nutrient components.

Bahwere et al. (2009) formulated a RUTF excluding NFDM while utilizing local ingredients in Malawi for malnourished HIV-positive adults that consisted of sesame seeds, chickpeas, corn, vegetable oil, sugar, and vitamins and minerals (CS-RUTF). Malnourished, HIV-positive females require 2600–2820 kcal and 48 g of protein to maintain weight, which is similar to the 2615 kcal needed by pregnant women, the target group in the present work, during the third trimester of pregna-ncy (Table2) (FAO, WHO, UNU 2001). The CS-RUTF contained 536.2 kcal/100 g energy and 12.3/100 g protein (Bahwere et al. 2009). When comparing the present RUTFs to the CSRUTF, the RUTF in this work has ∽1.50 to 1.61-fold more protein and ∽1.16 to 1.18-fold less energy.

The amino acid profiles of the 6 RUTFs are shown in Table5. The levels of several essential amino acids including lysine, histidine, threonine, and cysteine fell slightly below software-predicted values in all 6 products. However, the differences between predicted and actual amino acid contents were small, ranging from 0.03% to 0.25% of the formulations (Table5). Two products (C and F) also had a lower (0.01%) than the predicted tryp-tophan content, and product C also had a lower (0.01%) than the predicted valine content. The greatest differe-nce was observed with the predicted and actual lysine content of the 6 RUTFs, ranging from 0.17% to 0.25%. This was most likely due to loss of lysine during thermal processing.

**Table 5:** Software-predicted and amino acids of the RUTFs (on a dry weight basis)

	Formulations with rice koji												Formulations with barley koji					
	A			B			C			D			E			F		
Amino acids	Predicted values	Actual values	Absolute difference	Predicted values	Actual values	Absolute difference	Predicted values	Actual values	Absolute difference	Predicted values	Actual values	Absolute difference	Predicted values	Actual values	Absolute difference	Predicted values	Actual values	Absolute difference
Lys	0.77	0.59	−0.17	0.77	0.57	−0.20	0.77	0.52	−0.25	0.77	0.56	−0.21	0.77	0.54	−0.22	0.77	0.52	−0.25
Leu	1.34	1.42	0.08	1.28	1.34	0.06	1.22	1.25	0.03	1.36	1.45	0.09	1.32	1.39	0.07	1.28	1.35	0.07
Val	0.80	0.82	0.02	0.79	0.79	0.01	0.78	0.77	−0.01	0.80	0.83	0.03	0.80	0.80	0.00	0.79	0.80	0.01
His	0.47	0.41	−0.06	0.46	0.40	−0.06	0.46	0.39	−0.07	0.47	0.42	−0.05	0.47	0.40	−0.07	0.46	0.39	−0.07
Trp	0.19	0.20	0.01	0.18	0.19	0.00	0.18	0.18	−0.01	0.19	0.19	0.00	0.19	0.19	0.00	0.20	0.18	−0.01
Thr	0.62	0.58	−0.04	0.61	0.58	−0.03	0.60	0.55	−0.05	0.62	0.58	−0.04	0.62	0.57	−0.05	0.62	0.57	−0.05
Ile	0.67	0.71	0.05	0.65	0.69	0.03	0.64	0.67	0.02	0.67	0.72	0.05	0.66	0.71	0.04	0.66	0.69	0.04
Met	0.25	0.27	0.02	0.24	0.26	0.02	0.24	0.25	0.01	0.25	0.28	0.03	0.25	0.26	0.01	0.25	0.26	0.01
Cys	0.23	0.21	−0.03	0.23	0.20	−0.03	0.22	0.19	−0.03	0.24	0.21	−0.03	0.24	0.21	−0.03	0.24	0.21	−0.03
Met + Cys	0.48	0.47	−0.01	0.47	0.46	−0.01	0.46	0.44	−0.03	0.49	0.49	0.00	0.49	0.47	−0.02	0.49	0.47	−0.02
Phe	0.95	1.00	0.05	0.94	0.98	0.04	0.92	0.94	0.02	0.96	1.02	0.06	0.96	1.00	0.04	0.95	0.99	0.04
Tyr	0.65	0.76	0.11	0.64	0.74	0.10	0.64	0.72	0.08	0.65	0.76	0.11	0.65	0.74	0.09	0.62	0.74	0.10
Phe + Tyr	1.60	1.76	0.16	1.58	1.72	0.14	1.56	1.65	0.10	1.61	1.78	0.17	1.61	1.73	0.13	1.60	1.73	0.13

The formulation software created nutritious formulae that satisfied the protein, and 5–7 essential amino acids, required by pregnant women (Tables5), showing that cereals and legumes can be used for the development of nutrient dense foods without the use of NFDM. However, the exclusion of NFDM from the current RUTFs had an impact on the protein and amino acid profiles, specifically the lysine content of the RUTFs because of the high content of protein and lysine—36.2 g of protein and 2.68 g of lysine (USDA 2012). The elimination of NFDM may also influence the protein quality of the RUTFs. Although plant proteins are lower in quality than animal, a high-quality protein mixture can be achieved solely using plant ingredients if complementary proteins are combined (Young and Pellett 1994). In the present research, legumes, peanuts and cowpeas, and cereals, millet, rice and barley, were mixed to provide a balanced, complete protein.

Protein and amino acid profiles of the current RUTFs could be improved through the inclusion of other higher quality plant ingredients such as soybeans, 100 g of which contain 36.5 g of protein and 2.71 g of lysine (USDA 2012). Soybean production is currently limited in Mali although Nigeria, another country in West Africa, had African soybean production in 2007 (Torheim et al. 2004; Soy Info Center 2013). The potential of using soybeans to improve the nutritional profile of the present RUTFs as well as the impact on total ingredient price needs to be further explored. Low lysine levels in the RUTFS could also be overcome through supplementa-tion with lysine HCl before or after processing to impr-ove the amino acid profile of the RUTFs (Rosenberg and Rohdenburg 1952).

In addition to the choice of ingredients, processing conditions could also have an impact on the contents of essential amino acids. Chemical reactions, specifically Maillard browning during processing, may occur which can reduce the overall amino acid contents of the products (Ames 1990). In the present study, the RUTFs were heated at 121°C for 15 min, which could have induced Maillard browning and contributed to the variations between actual and software-predicted amino acid values. However, the thermal process used in the present study is essential for the production of safe and microbiologically stable products.

### Calculated amount of RUTFs needed to meet daily nutrient requirements

**Figure 1:**
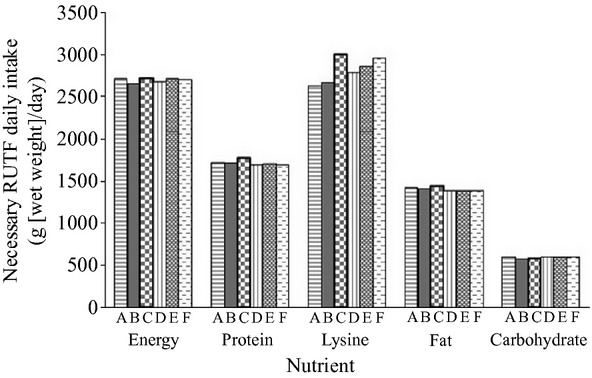
The amount of each ready-to-use therapeutic foods (RUTF) (A–F, shown from left to rigth) that must be consumed to meet the energy, protein, lysine (most limiting amino acid), fat, and carbohydrate requirements, based on the assumption that the RUTFs are the only source of nutrient in pregnant women's diet. The reference nutrient requirements for pregnant women in their 3rd trimester are: energy – 2615.20 kcal; protein – 64.00 g; lysine – 3.62 g; fat – 58.12 g; and carbohydrates – 65.00 g (Table2).

In order to meet the daily requirement of carbohydrate, fat and protein shown in Table2 as the sole source of nutrients, approximately 578–610 g, 1389–1443 g, or 1703–1784 g (wet weight) of RUTFs will have to be consumed. To meet the requirements for energy or lysine (Table2) approximately 2652–2713 g or 2620–3002 g will have to be consumed (Fig.1). Since lysine is the most limiting nutrient in all 6 RUTFs, consuming an adequate amount of products to meet the requirement of lysine will also supply sufficient amounts of other macronutrients.

RUTFs developed by Nabuuma et al. (2012) targeted young children and approximately 1257–1386 g would be required per day to provide the 1400 kcal/day of energy needed by a 15.4 lb child (7 kg). However, the amount of product that can be consumed per day by a patient is dependent on the severity of malnutrition and a person's willingness to consume the product. In a 3-month clinical trial in Malawi, HIV-positive patients with a BMI indicative of malnutrition were given 500 g/day of the CS-RUTF described above (Bahwere et al. 2009). However, an average daily intake of 300 g was recorded (Bahwere et al. 2009). Even with lower than expected daily intake, weight gain was observed in 73.3% of the participants (Bahwere et al. 2009). This suggests that weight gain can be accomplished even if a portion of the product is consumed. The amount of necessary RUTF consumption can be adjusted based on a patient's needs, allowing treatment for malnutrition to be easily customized with the same formula.

### Price trend of RUTFs

Table6 shows predicted ingredient costs to produce 1 kg of the RUTF products based on the average yearly ingredient price during the period 2004–2009. It is observed that the costs for producing any of the 6 RUTFs increased from $0.33/kg dry product in 2004 to $0.50/kg dry product in 2009 with a total change of $0.17/kg dry product due to the rise in ingredient prices (Table6).

**Table 6:** Changes in ingredient costs ($US/kg of dry product) of RUTF production due to increases in commodity prices in Mali from 2004 to 2009

	RUTF ingredient price in US $/kg of dry product						
Year	A	B	C	D	E	F	Average
2004	0.33	0.34	0.34	0.33	0.33	0.33	0.33 ± 0.006
2005	0.36	0.36	0.36	0.36	0.35	0.35	0.36 ± 0.005
2006	0.37	0.38	0.38	0.37	0.37	0.37	0.37 ± 0.006
2007	0.41	0.42	0.43	0.41	0.41	0.41	0.42 ± 0.006
2008	0.46	0.46	0.47	0.45	0.46	0.46	0.46 ± 0.006
2009	0.49	0.50	0.50	0.49	0.50	0.50	0.50 ± 0.003

However, the price for producing RUTFs includes the costs of both processing and ingredients. UNICEF reports showed that 68% of the overall cost of PlumpyNut® comes from ingredient purchases (Katzman 1956). The price of imported PlumpyNut® in Kenya in 2008 was approximately $5.00/kg (Duke University 2009). This suggests that ∽$3.40/kg of the final price of the imported PlumpyNut® was spent on ingredients (Katzman 1956; Duke University 2009). Sandige et al. (2004) compared the price of PlumpyNut® imported into Malawi with a locally produced RUTF containing NFDM. The imported PlumpyNut® was $5.00/kg, including shipping and duty, while the cost of the locally produced RUTF with the similar ingredients was $1.25/kg, resulting in a difference of $3.75/kg (Sandige et al. 2004). The RUTF in the present work, which does not contain NFDM, is ∽$0.75–$0.92/kg less than the locally produced RUTF in Malawi. Regardless of the processing costs, reducing the expenses on ingredients will significantly reduce the final cost of a RUTF (Katzman 1956; Duke University 2009).

Using plant-based ingredients to develop and RUTF for the region of East Africa, Dibari et al. (2012) used maize, soy, sorghum, palm olein oil, and sugar resulting in a formulation that was ∽$0.70/kg. The ingredient costs for the present RUTFs containing plant-based ingredients ranged from $0.33 to $0.50/kg dry product which, along with previous research, suggests that even with added processing and packaging costs the total production cost for the current RUTFs is likely to be substantially lower than that of the PlumpyNut® (Dibari et al. 2012).

## Conclusion

Formulation computer software can be utilized to develop nutrient dense, cost effective RUTFs based largely on local, plant-based ingredients for malnourished populations, in this case pregnant women in Mali. The actual protein, fat, ash, fiber, and amino acid contents of the developed RUTFs were comparable with computer software-predicted values. Energy contents of the RUTFs were 14.8–22.2% higher than predicted values, providing a higher energy density per 100 g dry basis of RUTF than originally expected. If the RUTFs are the only source of nutrients for a malnourished pregnant woman, 2620–3002 g of RUTFs are needed in order to meet the daily requirement for the most limiting nutrient, lysine, or energy. However, the amount of RUTF needed to meet daily nutrient requirements can be adjusted, depending on the other sources of nutrients in the diet. The fat and energy contents of the products can be increased through the use of oils and/or changing the restriction level for fat content. Once fortified with vitamins and minerals, the RUTFs will satisfy the overall nutrient requirement of pregnant women in their third trimester. The costs for producing the present RUTFs varied each year during the period 2004–2009 due to increases in ingredient prices but were substantially lower than imported commercial RUTFs.
